# Several Basic Elements of Entropic Statistics

**DOI:** 10.3390/e25071060

**Published:** 2023-07-13

**Authors:** Zhiyi Zhang

**Affiliations:** Department of Mathematics and Statistics, UNC Charlotte, Charlotte, NC 28223, USA; zzhang@charlotte.edu

**Keywords:** entropies, entropy estimation, entropic-moment-generating function, entropic statistics

## Abstract

Inspired by the development in modern data science, a shift is increasingly visible in the foundation of statistical inference, away from a real space, where random variables reside, toward a nonmetrized and nonordinal alphabet, where more general random elements reside. While statistical inferences based on random variables are theoretically well supported in the rich literature of probability and statistics, inferences on alphabets, mostly by way of various entropies and their estimation, are less systematically supported in theory. Without the familiar notions of neighborhood, real or complex moments, tails, et cetera, associated with random variables, probability and statistics based on random elements on alphabets need more attention to foster a sound framework for rigorous development of entropy-based statistical exercises. In this article, several basic elements of entropic statistics are introduced and discussed, including notions of general entropies, entropic sample spaces, entropic distributions, entropic statistics, entropic multinomial distributions, entropic moments, and entropic basis, among other entropic objects. In particular, an entropic-moment-generating function is defined and it is shown to uniquely characterize the underlying distribution in entropic perspective, and, hence, all entropies. An entropic version of the Glivenko–Cantelli convergence theorem is also established.

## 1. Introduction and Summary

Let $X=\{\ell_{k};k\geq 1\}$ be a countable alphabet and let $p=\{p_{k};k\geq 1\}$ be a probability distribution on X. Let P be the collection of all probability distributions on X. Let $p_{↓}=\left\{p_{\left(k\right)};k\geq 1\right\}$ be the nonincreasingly rearranged p, that is, $p_{\left(k\right)}\geq p_{\left(k+1\right)}$ for every k≥1. Let $P_{↓}$ be the collection of all possible $p_{↓}$. It follows that $P_{↓}\subset P$ is an aggregated version of P in the sense that P is partitioned and represented by $p_{↓}\in P_{↓}$.

Across a wide spectrum of scientific investigation, a random system is often described as a probability distribution on a countable alphabet, {X,p}; however many complex system properties of interest, such as those studied in information theory and statistical mechanics, are often described by functions of $p_{↓}$, for example, the Shannon entropy

$$H=-\sum_{k\geq 1}p_{k}\ln p_{k}$$

as in [1], the members of the Rényi entropy family

$$R_{\alpha }=\ln\sum_{k\geq 1}p_{k}^{\alpha }/\left(1-\alpha \right)$$

where α∈(0,1)∪(1.∞), as in [2], and the members of the Tsallis entropy family

$$T_{\alpha }=\left(1-\sum_{k\geq 1}p_{k}^{\alpha }\right)/\left(\alpha -1\right)$$

where α∈(−∞,1)∪(1,∞), as in [3]. Other similar functions come under the names of diversity indices, for example, the Gini–Simpson index

$$\zeta =1-\sum_{k\geq 1}p_{k}^{2}$$

as in [4], the generalized Simpson’s indices

$$\zeta_{u,v}=\sum_{k\geq 1}p_{k}^{u}{\left(1-p_{k}\right)}^{v}$$

where u≥1 and v≥0 are integers, as described in [5], Hill’s diversity numbers

$$H_{\alpha }={\left(\sum_{k\geq 1}p_{k}^{\alpha }\right)}^{1/(1-\alpha )}$$

where α∈(0,1)∪(1,∞), as in [6], Emlen’s index

$$D=\sum_{k\geq 1}p_{k}e^{-p_{k}},$$

as in [7], and the richness index

$$K=\sum_{k\geq 1}1\left[p_{k}>0\right],$$

where 1[·] is the indicator function. While all the abovementioned functions each have their unique significance in their respective fields of study, they share one characteristic in common: they are all functions of $p_{↓}$.

The word *entropy* has ancient Greek roots, *en* and *tropē*, that is, inward and change respectively, in English, or internal change collectively. As such, it is a label-independent concept. For generality and conciseness of the presentation in this article, let the following definition be adopted.

**Definition** **1.** *Let f(p) be a function defined for every p∈P. The function f(p) is referred to as an entropy if f(p) depends on p only through $p_{↓}$, that is, $f\left(p\right)=f\left(p_{↓}\right)$.*

By Definition 1, all entropies and diversity indices mentioned about are indeed entropies. In addition, $p_{\left(1\right)}$, or more generally $p_{\left(k\right)}$ for any positive integer *k*, is an entropy, and therefore $p_{↓}$ is an array of entropies. One important property to be noted about entropies is that $p_{↓}$ is independent of labels of the alphabet, $\left\{\ell_{k};k\geq 1\right\}$. Another fact to be noted is that all entropies are uniquely determined by $p_{↓}$. For clarity of terminologies throughout this article, let it be noted that any properties of the underlying random system that are described by one or more entropies are referred to as entropic properties. Furthermore, p is referred to as the underlying probability distribution, or simply the distribution, of a random system, and $p_{↓}$ is referred to as the entropic distribution associated with p. It is also to be noted that $p_{↓}=\left\{p_{\left(k\right)};k\geq 1\right\}$ is not a probability distribution in the usual sense since it is not associated with any specific probability experiment. It is merely an array of nonincreasingly ordered positive parameters that sum up to one.

Let $\left\{X_{1},\cdots ,X_{n}\right\}$, drawn from X according to p, be a random sample of size *n*. The sample may be summarized into $Y=\{Y_{k};k\geq 1\}$, where $Y_{k}$ is the observed frequency of letter $\ell_{k}$, or into $\hat{p}=\left\{{\hat{p}}_{k}=Y_{k}/n;k\geq 1\right\}$. Let $Y_{↓}=\left\{Y_{\left(k\right)};k\geq \right\}$ and ${\hat{p}}_{↓}=\left\{{\hat{p}}_{\left(k\right)}=Y_{\left(k\right)}/n;k\geq 1\right\}$ be the nonincreasingly rearranged Y and $\hat{p}$, respectively, where $Y_{\left(k\right)}\geq Y_{\left(k+1\right)}$ and ${\hat{p}}_{\left(k\right)}\geq {\hat{p}}_{\left(k+1\right)}$ for every *k*. Under the assumption that the study interest of the underlying random system only lies with the properties described by indices that are functions of the form $f(p_{↓})$, that is, entropies by Definition 1, there are two conceptual perspectives to the associated with statistical inference. The first is a framework of estimating f(p) based on $\hat{p}$, and the second is one of estimating $f\left(p\right)=f\left(p_{↓}\right)$ based on ${\hat{p}}_{↓}$. For lack of better terms, let the first framework be referred to as the classical statistics and the second framework as the entropic statistics. These two frameworks are not equivalent and, in particular, the entropic framework has its special and useful implications.

The literature of statistical estimation of entropies, mostly in the specific form of the Shannon entropy, begins with the early works, as in [8,9,10], and expands in width and depth in works by, for example, [11,12,13]. Many other worthy references on entropy estimation may be found in the literature review in [14]. The general entropies of Definition 1, however, allow a discussion on the foundational elements of the statistics in entropic perspective, or entropic statistics, in a broader sense. This article focuses on three basic basic issues.

First, a notion of entropic sample space is introduced in Section 2 below. An entropic sample space is an aggregated sample space to register; not a single data point, but an ensemble of data points. It is a sample space of the entropic statistics, $Y_{↓}$ or ${\hat{p}}_{↓}$, and hence is label-independent. The said label-independence in turn allows an entropic sample space to accommodate statistical sampling into a population that is not necessarily prescribed, that is, the labels of alphabet X need not be completely specified *a priori*. This property of an entropic sample space gives new meaning to statistical learning and lends foundational support for statistical exploration into an unknown, or partially known, universe.

Second, an entropic characteristic function, $\phi \left(t\right)=\sum_{k\geq 1}p_{\left(k\right)}^{t}$ for t≥1, is introduced. It is obvious that ϕ(t) is an entropy by Definition 1 and that it always exists. It is established in Section 3 that ϕ(t) in an arbitrarily small neighborhood of any interior point of [1,∞) uniquely determines the $p_{↓}\in P_{↓}$ and *vice versa*. Therefore, it is immediately implied that any and all entropic properties of a random system, including statistical inferences, may be approached by way of ϕ(t).

Third, it is established in Section 4 that the entropic statistics converges almost surely and uniformly to the underlying entropic distribution, that is, ${\hat{p}}_{↓}\overset{a.s.}{⟶}p_{↓}$ uniformly, for any $p_{↓}\in P_{↓}$. In light of the entropic sampling space and an entropic characterization of the associated entropic sampling distribution, the Glivenko–Cantelli-like convergence theorem provides a fundamental support in theory for exercises in entropic statistics.

The article ends with an appendix where a lengthy proof is found.

## 2. Things Entropic

### 2.1. Sample Spaces in Different Resolutions

Consider the experiment of randomly drawing a marble from urn 1, which contains marbles of K=3 known colors, *red*, *white*, and *blue*. In anticipating the outcome of the experiment, one may introduce an index *k*, k=1,2,3, to label the possible outcomes by $\ell_{1}=\mathrm{red}$, $\ell_{2}=\mathrm{white}$, and $\ell_{3}=\mathrm{blue}$, and denote the corresponding proportions by $p_{1}$, $p_{2}$, and $p_{3}$. In this case, the sample space is

(1) $$\Omega_{1}=\left\{\ell_{1},\ell_{2},\ell_{3}\right\},$$

the event space is $B=\{\emptyset ,\left\{\ell_{1}\right\},\left\{\ell_{2}\right\},\left\{\ell_{3}\right\},\left\{\ell_{1},\ell_{2}\right\},\left\{\ell_{1},\ell_{3}\right\},\left\{\ell_{2},\ell_{3}\right\},\left\{\ell_{1},\ell_{2},\ell_{3}\right\}\}$, and the point mass probability measure μ(·) assigns $p_{1}$ to $\ell_{1}$, $p_{2}$ to $\ell_{2}$, and $p_{3}$ to $\ell_{3}$. Let *X* denote the random outcome of the experiment. The following model of probability distribution,

(2) $$\begin{array}{cccc} \;X\; & \;\ell_{1}\; & \;\ell_{2}\; & \;\ell_{3}\;\\ P(x) & p_{1} & p_{2} & p_{3} \end{array}$$

or in a different form $p=\{p_{1},p_{2},p_{3}\}$ on $X=\Omega_{1}=\left\{\ell_{1},\ell_{2},\ell_{3}\right\}$, is well defined with three parameters, $p_{1}$, $p_{2}$, and $p_{3}$, subject to the constraints, $0\leq p_{k}\leq 1$ for each *k* and $\sum_{k=1}^{3}p_{k}=1$. The result of drawing n=1 marble from the urn may also be represented by a triplet of random variables $Y=\{1\left[X=\ell_{1}\right],1\left[X=\ell_{2}\right],1\left[X=\ell_{3}\right]\}$. If Y is used to represent the outcome of the experiment, the sample space may be denoted as $\Omega_{1}=\left\{\left\{1,0,0\right\},\left\{0,1,0\right\},\left\{0,0,1\right\}\right\}$ with corresponding probability distribution $P\left(Y=\left\{1,0,0\right\}\right)=p_{1}$, $P\left(Y=\left\{0,1,0\right\}\right)=p_{2}$ and $P\left(Y=\left\{0,0,1\right\}\right)=p_{3}$. For clarity in terminology, *X* is referred to as a random element but Y is a set of random variables. In general, random results of an experiment that are represented by numerical values are referred to as *random variables*, and those by non-numerical symbols are *random elements*.

For a given experiment, the sample space may be chosen at different levels of resolution depending on the experimenter’s interest in the study. Suppose the experimenter is to randomly draw n=3 marbles from urn 1 with replacement in sequence, resulting in $X=\{X_{1},X_{2},X_{3}\}$ where $X_{i}$, i=1,2,3, is the color of the *i*th marble drawn in the sequence. The sample space associated with X may be represented by

(3) $$\Omega_{s}=\left\{\begin{array}{ccc} \{\ell_{1},\ell_{1},\ell_{1}\}, & \{\ell_{2},\ell_{2},\ell_{2}\}, & \{\ell_{3},\ell_{3},\ell_{3}\},\\ \{\ell_{1},\ell_{1},\ell_{2}\}, & \{\ell_{2},\ell_{1},\ell_{1}\}, & \{\ell_{1},\ell_{2},\ell_{1}\},\\ \{\ell_{1},\ell_{1},\ell_{3}\}, & \{\ell_{3},\ell_{1},\ell_{1}\}, & \{\ell_{1},\ell_{3},\ell_{1}\},\\ \{\ell_{2},\ell_{2},\ell_{1}\}, & \{\ell_{1},\ell_{2},\ell_{2}\}, & \{\ell_{2},\ell_{1},\ell_{2}\},\\ \{\ell_{2},\ell_{2},\ell_{3}\}, & \{\ell_{3},\ell_{2},\ell_{2}\}, & \{\ell_{2},\ell_{3},\ell_{2}\},\\ \{\ell_{3},\ell_{3},\ell_{1}\}, & \{\ell_{1},\ell_{3},\ell_{3}\}, & \{\ell_{3},\ell_{1},\ell_{3}\},\\ \{\ell_{3},\ell_{3},\ell_{2}\}, & \{\ell_{2},\ell_{3},\ell_{3}\}, & \{\ell_{3},\ell_{2},\ell_{3}\},\\ \{\ell_{1},\ell_{2},\ell_{3}\}, & \{\ell_{1},\ell_{3},\ell_{2}\}, & \{\ell_{2},\ell_{1},\ell_{3}\},\\ \{\ell_{2},\ell_{3},\ell_{1}\}, & \{\ell_{3},\ell_{1},\ell_{2}\}, & \left\{\ell_{3},\ell_{2},\ell_{1}\right\} \end{array}\right\},$$

where the subscript “*s*” stands for sequential. There are 27 distinct elements in (3). In this case, the sample space may also be expressed as $\Omega_{s}={\left\{\ell_{1},\ell_{2},\ell_{3}\right\}}^{3}$. This sample space may be adopted if the order of the n=3 observations is observable and is of interest.

Suppose in the above experiment the order of the observations is not observable or not of interest. Then the relevant information in $X=\{X_{1},X_{2},X_{3}\}$ may be represented in the form of $Y=\{Y_{1},Y_{2},Y_{3}\}$, where $Y_{k}$, k=1,2, and 3 is the number of $\ell_{k}$s observed in the sample. The sample space associated with Y is

(4) $$\Omega_{m}=\left\{\begin{array}{ccccc} \{3,0,0\}, & \{0,3,0\}, & \{0,0,3\}, & \{2,1,0\}, & \{2,0,1\},\\ \{0,2,1\}, & \{1,2,0\}, & \{0,1,2\}, & \{1,0,2\}, & \left\{1,1,1\right\} \end{array}\right\},$$

where the subscript “*m*” stands for multinomial. There are 10 distinct elements in (4). In fact, $Y=\{Y_{1},Y_{2},Y_{3}\}$ is the usual multinomial random vector with K=3 categories and category probabilities $p_{1}$, $p_{2}$, and $p_{3}$.

The two sample spaces, $\Omega_{s}$ and $\Omega_{m}$, serve different statistical interests in various situations. $\Omega_{s}$ is well defined if $X=\{X_{1},X_{2},X_{3}\}$ is observable. $\Omega_{m}$ is well defined if $X=\{X_{1},X_{2},X_{3}\}$ is observable or only $Y=\{Y_{1},Y_{2},Y_{3}\}$ is observable. Noting that $X=\{X_{1},X_{2},X_{3}\}$ implies $Y=\{Y_{1},Y_{2},Y_{3}\}$, a lower-resolution sample space may always be adopted if a higher-resolution sample space may, but not vice versa. For example, if the order of the draws is not observable, then only $\Omega_{m}$ is appropriate since $Y=\{Y_{1},Y_{2},Y_{3}\}$ is not linked uniquely to the elements of $\Omega_{s}$.

$\Omega_{m}$ is an aggregated form of $\Omega_{s}$ and is hence of lower resolution; however, $\Omega_{m}$ may be further reduced in resolution. Let

(5) $$Y_{↓}=\left\{Y_{\left(1\right)},Y_{\left(2\right)},Y_{\left(3\right)}\right\},$$

where $Y_{\left(1\right)},Y_{\left(2\right)},Y_{\left(3\right)}$ are nonincreasingly ordered observed frequencies of the three colors. The sample space associated with $Y_{↓}$ is

(6) $$\Omega_{e}=\left\{\begin{array}{ccc} \{3,0,0\}, & \{2,1,0\}, & \left\{1,1,1\right\} \end{array}\right\},$$

where the subscript “*e*” stands for entropic. $\Omega_{e}$ is yet an aggregated form of $\Omega_{m}$ and hence of lower resolution still than that of $\Omega_{m}$. Noting that $\Omega_{e}$ is label-independent, it is an example of entropic sample space.

It is easily verified that the probability distribution of $Y_{↓}$ may be expressed in terms of $p_{↓}$ as follows.

(7) $$\begin{array}{cccc} Y_{↓} & \left\{3,0,0\right\} & \left\{2,1,0\right\} & \left\{1,1,1\right\}\\ P(y_{↓}) & \sum_{k\geq 1}p_{\left(k\right)}^{3} & 3\sum_{k\geq 1}p_{\left(k\right)}^{2}\left(1-p_{\left(k\right)}\right) & 6p_{\left(1\right)}p_{\left(2\right)}p_{\left(3\right)} \end{array}$$

Let it be noted that all the probabilities in (7) are label-independent, and therefore they are entropies by Definition 1.

In the case of sampling n=3 marbles from urn 1 in sequence, a subscription to the entropic sample space, $\Omega_{e}$, is by choice since both $\Omega_{m}$ and $\Omega_{e}$ are available. There are situations when the subscription to an entropic sample space may be by necessity.

Consider the experiment of randomly drawing n=3 marbles in sequence from urn 2, which contains marbles of K=3 unknown but distinguishable colors. In this case, the sample spaces, $\Omega_{1}$ of (1) and $\Omega_{m}$ of (3), are not well defined due to the lack of knowledge of the color labels. However, the entropic sample space, $\Omega_{e}$, is available for subscription regardless of what the colors are, known or unknown, as long as they are distinguishable.

In general, consider drawing a random sample of size *n* from $X=\{\ell_{k};k\geq 1\}$ under $p=\{p_{k};k\geq 1\}$ in sequence. The sequential sample space is of the form $\Omega_{1}=X^{n}$. The aggregated sample space,

(8) $$\Omega_{m}=\left\{\left\{y_{k};k\geq 1\right\}:y_{k}\geq 0\;\mathrm{for}\;\mathrm{every}\;k\geq 1\;\mathrm{and}\;\sum_{k\geq 1}y_{k}=n\right\},$$

is that of the mutinomial array, $Y=\{Y_{k};k\geq 1\}$, with probability mass function

(9) $$P\left(\left\{y_{k};k\geq 1\right\}\right)=\frac{n!}{\prod_{k\geq 1}y_{k}!}\prod_{k\geq 1}p_{k}^{y_{k}}$$

where $0\leq y_{k}\leq n$ for every k≥0 and $\sum_{k\geq 1}y_{k}=n$. Moreover, $\Omega_{m}$ may be further aggregated into a sample space, $\Omega_{e}$, for $Y_{↓}=\left\{Y_{\left(k\right)};k\geq 1\right\}$, that is,

(10) $$\Omega_{e}=\left\{\left\{y_{\left(k\right)};k\geq 1\right\}:y_{\left(k\right)}\geq 0\;\mathrm{and}\;y_{\left(k\right)}\geq y_{\left(k+1\right)}\;\mathrm{for}\;\mathrm{every}\;k\geq 1,\;\mathrm{and}\;\sum_{k\geq 1}y_{\left(k\right)}=n\right\}.$$

Let $\Omega_{e}$ of (10) be referred to as the entropic sample space. The associated probability distribution is

(11) $$P\left(\left\{y_{\left(k\right)};k\geq 1\right\}\right)=\sum_{*}P\left(\left\{y_{k};k\geq 1\right\}\right)$$

where $\sum_{*}$ is summation of (9) over all $\left\{y_{k};k\geq 1\right\}$s in $\Omega_{m}$ sharing the same given $\left\{y_{\left(k\right)};k\geq 1\right\}$.

Given a $y_{↓}=\left\{y_{\left(k\right)};k\geq 1\right\}$, (11) is an entropy. This may be seen in two steps. First, let $\hat{K}=\sum_{k\geq 1}1\left[y_{\left(k\right)}\geq 1\right]$ be the number of distinct letters of X represented in a sample of size *n*, and let $z=\{z_{1},\cdots ,z_{\hat{K}}\}$ be the set of $\hat{K}$ positive integer values of $y_{↓}$. $\hat{K}$ is a positive finite integer. Let the cardinality of X be denoted as $K=\sum_{k\geq 1}1\left[p_{k}>0\right]$. K≥1 may be finite or countably infinite. Consider an array $a\left(y_{↓}\right)=\left\{a_{k}\left(y_{↓}\right);k\geq 1\right\}$ of length *K* whose entries are a particular allocation of the $\hat{K}$ values of $z_{j}$, $j=1,\ldots ,\hat{K}$, with the other $K-\hat{K}$ values of $a(y_{↓})$ being zeros. Let $A(y_{↓})$ be the complete collection of all such distinct $a(y_{↓})$s. Then it is clear that $y_{↓}$ uniquely implies $A(y_{↓})$.

Second, the probability in (11) may be re-expressed as

(12) $$P\left(\left\{y_{\left(k\right)};k\geq 1\right\}\right)=\frac{n!}{\prod_{k\geq 1}y_{\left(k\right)}!}\sum_{**}\left(\prod_{k\geq 1}p_{\left(k\right)}^{a_{k}\left(y_{↓}\right)}\right)$$

where $\sum_{**}$ is summation over all $a\left(y_{↓}\right)\in A\left(y_{↓}\right)$, given a $y_{↓}=\left\{y_{\left(k\right)};k\geq 1\right\}$. Equation (12) implies that $P(\left\{y_{\left(k\right)};k\geq 1\right\})$ is a function of $p_{↓}$ and hence an entropy. Let $P(\left\{y_{\left(k\right)};k\geq 1\right\})$ of (11) or (12) be referred to as the *entropic distribution* associated with the entropic sample space, $\Omega_{e}$.

### 2.2. Entropic Objects

Let the adjective “entropic” be used to describe objects that are label-independent. Several such objects are defined or summarized below.

- A function, $f\left(p\right)=f\left(p_{↓}\right)$ for all $p_{↓}\in P_{↓}$, is an entropy.
- The elements of $p_{↓}=\left\{p_{\left(k\right)};k\geq 1\right\}$ are the entropic parameters, as compared to the elements of $p=\{p_{k};k\geq 1\}$, which are multinomial parameters.
- The elements of $Y_{↓}=\left\{Y_{\left(k\right)};k\geq 1\right\}$ or equivalently of ${\hat{p}}_{↓}=\left\{{\hat{p}}_{\left(k\right)};k\geq 1\right\}$ are entropic statistics, as compared to the elements of $Y=\{Y_{k};k\geq 1\}$ or equivalently $\hat{p}=\left\{{\hat{p}}_{k};k\geq 1\right\}$, which are multinomial statistics.
- $\Omega_{e}$ of (10) is the entropic (multinomial) sample space, as compared to $\Omega_{m}$ of (8), which is the multinomial sample space.
- The distribution $P(\left\{y_{\left(k\right)};k\geq 1\right\})$ of (11) or (12), is the entropic probability distribution, while $P(\left\{y_{k};k\geq 1\right\})$ of (9) is the multinomial probability distribution.
- Entropic statistics is the collection of statistical methodologies that help to make inference on the characteristics of a random system exclusively via entropies.

In addition, there are several useful other entropic objects. First, letting $\zeta_{v}=\sum_{k\geq 1}p_{k}{\left(1-p_{k}\right)}^{v}$ for all non-negative integers v≥0, $\zeta =\{\zeta_{v};v\geq 0\}$ is referred to as the *entropic basis*. The name comes from the fact that, for any well-behaved function, h(p) for p∈[0,1], an entropy of the form $H=\sum_{k\geq 1}p_{k}h\left(p_{k}\right)$ may be expressed as a linear combination $H=\sum_{v\geq 1}w\left(v\right)\zeta_{v}$. For example, the Shannon entropy, provided that it is finite, may be written as

$$H=-\sum_{k\geq 1}p_{k}\ln p_{k}=\sum_{v\geq 1}\left(1/v\right)\zeta_{v-1}.$$

The entropic basis is useful because it unfolds many entropies into simple and linearly additive forms.

Second, letting $\eta_{u}=\sum_{k\geq 1}p_{k}^{u}$ for all positive integers u≥1, $\eta =\{\eta_{u};u\geq 1\}$ is often referred to as the entropic moment. The elements of both ζ and η have good estimators. A detailed discussion may be found in [14].

**Definition** **2.** *Let X be an random element on a countable alphabet $X=\{\ell_{k};k\geq 1\}$ with a corresponding probability distribution p∈P and its associated entropic distribution $p_{↓}\in P_{↓}$. The function,*

(13) $$\phi \left(t\right)=\sum_{k\geq 1}p_{k}^{t},\;for\;t\geq 0,$$

*is referred to as the entropic-moment-generating function of X, of p, or of $p_{↓}$. The two complementary parts of its domain, [1,∞) and [0,1), are, respectively, referred to as the primary domain and the secondary domain of the entropic-moment-generating function.*

Depending on context, ϕ(t) may be denoted as $\phi_{X}\left(t\right)$, $\phi_{p}\left(t\right)$, or $\phi_{p_{↓}}\left(t\right)$ whenever appropriate. Obviously, ϕ(t) is uniformly bounded above by one for all p∈P in the primary domain but is not necessarily finitely defined in the secondary domain. However, in the case of a finite alphabet, that is, $K=\sum_{k\geq 1}1\left[p_{k}>0\right]<\infty$, ϕ(t) is finitely defined for each and every t∈R, in particular for t≥0. The characteristic utility of ϕ(t) is further explored in Section 3 below.

### 2.3. Examples of Entropic Statistics

**Example** **1.** *Consider the Bernoulli experiment of tossing a coin, where P(h)=p and P(t)=1−p. The question of whether the coin is fair may be formulated in the usual classical sense, that is, whether p=0.5. The question may be approached by estimating p based on a sample proportion, $\hat{p}$, if it is observable which trials lead to “h” and which lead to “t”. The question may alternatively be formulated by an equivalent entropic statement, for example, whether H=p(1−p)=0.25. More generally, if $K=\sum_{k\geq 1}1\left[p_{k}\right]$ is finite and known, then the uniformity of p on X may be formulated entropically by, for example, $H=\sum_{k\geq 1}p_{k}^{2}=1/K$, $H=\sum_{k\geq 1}p_{k}\left(1-p_{k}\right)=\left(K-1\right)/K$, or $H=-\sum_{k\geq 1}p_{k}\ln p_{k}=\ln K$. The validity of these entropic statements may then be gauged statistically.*

**Example** **2.** *Consider a two-stage sampling scheme: a random sample of size n, $\left\{X_{1},\cdots ,X_{n}\right\}$, and then a single extra observation $X_{n+1}$ are taken. The sample of size n may be summarized into letter frequencies, $Y=\{Y_{k};k\geq 1\}$. Let $\pi_{0}=\sum_{k\geq 1}p_{k}1\left[Y_{k}=0\right]$. Clearly, $\pi_{0}$ is label-independent and therefore an entropic random variable. Given the sample of size n, $\pi_{0}$ may be thought of as the probability of that $X_{n+1}$ assumes a letter in X that is not represented in the sample of size n. In some context, $\pi_{0}$ may be thought of as the probability of new discovery. Let $N_{1}=\sum_{k\geq 1}1\left[Y_{k}=1\right]$ and $T_{n}=N_{1}/n$. $T_{n}$ is commonly known as Turing’s formula, introduced in [15], but credited largely to Alan Turing. It is to be noted that $N_{1}$ is label-independent and, therefore, so is $T_{n}$. $T_{n}$ is an good estimator of $\pi_{0}$ and a discussion on many of its statistical properties may be found in [14].*

**Example** **3.** *In developing a decision tree classifier, the data space is partitioned into an ensemble of small subspaces, in each of which a local classification rule is sought. The central spirit of every local classification may be described by a two-step scheme.*

*1.*  *First, a random sample of size n, $\left\{X_{1},\cdots ,X_{n}\right\}$, is taken from $X=\{\ell_{k};k\geq 1\}$, under an unknown $p=\{p_{k};k\geq 1\}$, which is summarized into $Y=\{Y_{k};k\geq 1\}$.*
*2.*  *The data-based local classification rule is as follows: the next observation, $X_{n+1}$, is predicted to be the letter which is observed most frequently in the sample of size n. For simplicity, let it be assumed that $p_{\left(1\right)}>p_{\left(2\right)}$, and a letter with the sample maximum frequency is unique (if not, some randomization may be employed).*

*Obviously, the designated letter based on a sample is not necessarily the letter associated with the letter corresponding to the maximum of $p_{k}$s. In such a setup, the performance of the tree classifier may be gauged by evaluating (calculating or estimating) the probability of the event that “the designated letter is the same letter of X with probability $p_{\left(1\right)}$”, that is,*

(14) $$P\left(\underset{\ell_{k};k\geq 1}{arg max}\left\{p\left(\ell_{k}\right);k\geq 1\right\}=\underset{\ell_{k};k\geq 1}{arg max}\left\{\hat{p}\left(\ell_{k}\right);k\geq 1\right\}\right).$$

*Note that the event in (14) is label-independent and hence the probability is an entropy, which may be estimated. The probability in (14) may reasonably called the confidence level of the simple classifier.*

*For illustration purpose, consider the special case of a binary X, with n=2m+1 for some positive integer m. For simplicity, n is chosen to be odd here so that $Y_{\left(1\right)}>Y_{\left(2\right)}$ always holds true. Suppose that $p_{1}=p_{\left(1\right)}>p_{\left(2\right)}=1-p_{\left(1\right)}$. The event that a classifier based on the sample of n correctly identifies the letter of maximum probability may be equivalently expressed as $Y_{1}\geq m+1$. The probability of such an event, (14), is*

(15) $$\begin{array}{cc} P\left(\ell_{1}=\underset{\ell_{1},\ell_{2}}{arg max}\left\{{\hat{p}}_{1},1-{\hat{p}}_{1}\right\}\right)\; & =P(Y_{1}\geq m+1)\\ \; & =\sum_{y\geq m+1}\frac{n!}{y!(n-y)!}p_{\left(1\right)}^{y}{\left(1-p_{\left(1\right)}\right)}^{n-y}, \end{array}$$

*which is independent of the assumption that $p_{1}>p_{2}=1-p_{1}$ and, therefore, is an entropy. More specifically, (15) is computed for several combinations of n and $p_{↓}$ and the resulting values are tabulated in Table 1. Table 1, and its likes, may be used in two different ways. First, given a fixed $p_{↓}$, it indicates how large a sample is needed to assure a reliability level of the classifier. On the other hand, at a given level of n and a particular $p_{↓}$, the classifier may be evaluated by the probabilities in the table. In practice, $p_{↓}$ is unknown but may be estimated.*

## 3. Entropic Characterization

Entropic statistics focuses on making inference via entropies; it is therefore of interest to find a function which may characterize $p_{↓}\in P_{↓}$. Since the function ϕ(t) in its primary domain and $\eta =\{\eta_{u};u\geq 1\}$, where $\eta_{u}=\sum_{k\geq 1}p_{k}^{u}$ and *u* is an integer, imply each other (see Lemma 1 below), it follows immediately that (13) uniquely determines all entropies. However, the following theorem claims that the characteristic property of the entropic-moment-generating function, ϕ(t), remains intact in any arbitrarily neighborhood of any t∈(1,∞).

**Theorem** **1.** *Let $p=\{p_{k};k\geq 1\}$ and $q=\{q_{k};k\geq 1\}$ be two probability distributions on a same countable alphabet, $X=\{\ell_{k};k\geq 1\}$. Let $p_{↓}=\left\{p_{\left(k\right)};k\geq 1\right\}$ and $q_{↓}=\left\{q_{\left(k\right)};k\geq 1\right\}$ be the respective corresponding entropic distributions of p and q. Then $p_{↓}=q_{↓}$ if and only if $\phi_{p}\left(t\right)=\phi_{q}\left(t\right)$ for all t∈(a,b) where (a,b) is an arbitrary interval such that 1≤a<b<∞.*

**Lemma** **1.** *Let $p=\{p_{k};k\geq 1\}$ and $q=\{q_{k};k\geq 1\}$ be two probability distributions in P with two corresponding associated entropic distributions $p_{↓}$ and $q_{↓}$ in $P_{↓}$. Then $p_{↓}=q_{↓}$ if and only if $\sum_{k\geq 1}p_{k}^{n}=\sum_{k\geq 1}q_{k}^{n}$ for all positive integers n≥1.*

A proof of Lemma 1 may be found on pages 50 and 51 in [14]. To prove Theorem 1, it suffices to show that ϕ(t) in an arbitrarily small neighborhood of any interior point of [1,∞) determines the function globally.

**Proof** **of** **Theorem** **1.** If $p_{↓}=q_{↓}$, then it immediately follows that $\phi_{p}\left(t\right)=\phi_{q}\left(t\right)$ for all t∈[1,∞) and, therefore, for t∈(a,b) specifically. To prove the theorem, it suffices to show the converse.

Consider the series

$$f\left(z\right)=\sum_{k=1}^{\infty }p_{k}^{z}$$

where z∈C is a complex variable. Denote the real and the imaginary parts of a complex value *z* by Re(z) and Im(z), respectively.

Let D={z:Re(z)>1} be the subset of C such that the real part of *z* is greater than 1. For every z∈D, since pkRe(z−1)≤1 and pkiIm(z)=1, for every *k*, where |z| is the modulus of *z*, it follows that

f(z)=∑k=1∞pkpkz−1=∑k=1∞pkpkRe(z−1)pkiIm(z)

and

(16) $$\left|f\left(z\right)\right|\leq \sum_{k=1}^{\infty }p_{k}.$$

Letting $\alpha_{k}=\ln\left(1/p_{k}\right)$,

(17) $$f\left(z\right)=\sum_{k=1}^{\infty }e^{-\alpha_{k}z},$$

and the functions $e^{-\alpha_{k}z}$, k≥1, are analytic on C.

Since the series in (17), for z∈D, is dominated by the convergent series $\sum_{k\geq 1}p_{k}$ as in (16), by the Weierstrass uniform convergence theorem, f(z) is analytic on D. By a similar argument, $g\left(z\right)=\sum_{k=1}^{\infty }q_{k}^{z}$ is also analytic on D.

Assuming that $\phi_{p}\left(t\right)=\phi_{q}\left(t\right)$ for t∈(a,b) where 1≤a<b<∞, there exists a convergent sequence, $\left\{z_{n};n\geq 1\right\}$ in (a,b) such that $\lim_{n\to \infty }z_{n}=z_{0}\in \left(a,b\right)$. Noting that (a,b)⊂D and $f\left(z_{n}\right)=\phi_{p}\left(z_{n}\right)=\phi_{q}\left(z_{n}\right)=g\left(z_{n}\right)$ for n≥0, by the identity theorem for analytic functions, f(z)=g(z) for all z∈D. It follows that $\phi_{p}\left(t\right)=f\left(t\right)=g\left(t\right)=\phi_{q}\left(t\right)$ for all t∈[1,∞), specifically, $\sum_{k\geq 1}p_{k}^{n}=\sum_{k\geq 1}q_{k}^{n}$ for all n≥1. Finally, by Lemma 1, $p_{↓}=q_{↓}$. □

Theorem 1 immediately implies that a subfamily of the Rényi entropy $R_{\alpha }$ with α∈(a,b)⊂(1,∞), a subfamily of the Tsallis entropy $T_{\alpha }$ with α∈(a,b)⊂(1,∞), and a subfamily of Hill’s diversity numbers $H_{\alpha }$ with α∈(a,b)⊂(1,∞), respectively, characterizes $p_{↓}$ and, hence, characterizes all entropies.

The characterization of $p_{↓}$ in Theorem 1 may be equivalently stated only on any infinitely countable subset of (a,b).

**Corollary** **1.** *Let p and q be two probability distributions on a same countable alphabet, X. Let $p_{↓}$ and $q_{↓}$ be the corresponding entropic distributions of p and q, respectively. Then $p_{↓}=q_{↓}$ if and only if $\phi_{p}\left(t\right)=\phi_{q}\left(t\right)$ on any infinite sequence of distinct values, $\left\{t_{n};n\geq 1\right\}$, such that $\lim_{n\to \infty }t_{n}=c\in \left(1,\infty \right)$.*

**Proof.**  Both $\phi_{p}\left(t\right)$ and $\phi_{q}\left(t\right)$ are analytic at t=c, and therefore $h\left(t\right)=\phi_{p}\left(t\right)-\phi_{q}\left(t\right)$ is analytic at t=c. Let it be first shown, by induction, that all derivatives of h(t) at t=c are zero, that is, $h^{\left(m\right)}\left(c\right)=0$ for m≥0. Note first that $h\left(c\right)=h^{\left(0\right)}=0$ by the fact that both $\phi_{p}\left(t\right)$ and $\phi_{q}\left(t\right)$ are continuous and $\lim_{n\to \infty }\phi_{p}\left(t_{n}\right)=\phi_{p}\left(c\right)=\phi_{q}\left(c\right)=\lim_{n\to \infty }\phi_{q}\left(t_{n}\right)$. Suppose that $h^{\left(0\right)}\left(c\right)=h^{\left(1\right)}\left(c\right)=h^{\left(2\right)}\left(c\right)=\cdots =h^{\left(m\right)}\left(c\right)=0$ but $h^{\left(m+1\right)}\left(c\right)\neq 0$. Then there exists an interval (c−ε,c+ε) such that h(t)≠0 for t∈(c−ε,c+ε). However, there is at least one $t_{n}\in \left(c-\varepsilon ,c+\varepsilon \right)$ such that $h(t_{n})=0$ by assumption. This is a contradiction and therefore $h^{\left(m\right)}\left(c\right)=0$ for all m≥1. □

**Corollary** **2.** *Let p and q be two probability distributions on a same countable alphabet, X. Let $p_{↓}$ and $q_{↓}$ be the corresponding entropic distributions of p and q, respectively. Then $p_{↓}=q_{↓}$ if and only if $\phi_{p}\left(t\right)=\phi_{q}\left(t\right)$ on any infinite sequence of distinct values, $\left\{t_{n};n\geq 1\right\}\in \left(a,b\right)$ where 1≤a<b<∞.*

**Proof.**  Noting that the infinitely many $t_{n}$s are in an bounded interval, there exists an infinite subset of $\left\{t_{n};n\geq 1\right\}$ that converges to a constant c∈[a,b]. The corollary follows Corollary 1. □

Consider a pair of random elements, (X,Y), on a countable joint alphabet, $X\times Y=\{\left(l_{i},m_{j}\right);i\geq 1,j\geq 1\}$, with a corresponding joint probability distribution, $p_{X,Y}=\left\{p_{i,j};i\geq 1,j\geq 1\right\}$. Let $p_{X}=\left\{p_{i,\cdot };i\geq 1\right\}$ and $p_{Y}=\left\{p_{\cdot ,j};j\geq 1\right\}$, where $p_{i,\cdot }=\sum_{j\geq 1}p_{i,j}$ and $p_{\cdot ,j}=\sum_{i\geq 1}p_{i,j}$, be the two marginal probability distributions of *X* and *Y*, respectively.

**Corollary** **3.** *X and Y are independent if and only if*

(18) $$\phi_{X,Y}\left(t\right)=\phi_{X}\left(t\right)\times \phi_{Y}\left(t\right)$$

*for all t∈(a,b), where a and b are two arbitrary real numbers such that 1≤a<b<∞.*

**Proof.**  If *X* and *Y* are independent, then (18) follows immediately. Conversely, suppose that (18) holds. Consider another pair of independent random elements, (U,V), on the same countable joint alphabet X×Y and with identical marginal distributions to those of (X,Y), that is, $p_{X}$ and $p_{Y}$. It then follows, by (18) and Theorem 2, that $p_{U,V}=p_{X,Y}$, which in turn implies that *X* and *Y* are independent. □

Corollary 3 provides a characterization of independence on a general countable joint alphabet, and its utility may be explored further.

## 4. A Basic Convergence Theorem

From an entropic perspective, the convergence of ${\hat{p}}_{↓}$ to $p_{↓}$, to be distinguished from that of $\hat{p}$ to p, is of fundamental interest.

For clarity of presentation in this section, let it be noted that, whenever necessary, the subindex *n* may be added to Y, $Y_{k}$, $\hat{p}$, ${\hat{p}}_{k}$, ${\hat{p}}_{↓}$, and ${\hat{p}}_{\left(k\right)}$ to highlight the dynamic nature of these previously defined quantities as *n* changes, that is, $Y=Y_{n}$, $Y_{k}=Y_{k,n}$, $\hat{p}={\hat{p}}_{n}$, ${\hat{p}}_{k}={\hat{p}}_{k,n}$, ${\hat{p}}_{↓,n}={\hat{p}}_{↓}$ and ${\hat{p}}_{\left(k\right)}={\hat{p}}_{(k),n}$, respectively.

The main result established in this section is the uniform almost-sure convergence of ${\hat{p}}_{↓}$ to $p_{↓}$, which is made more precise in Theorem 2 below.

Consider the experiment of repeatedly and independently drawing a letter from X under p, resulting in a sequence of randomly selected letters, $\omega =\{x_{1},x_{2},\cdots \}$. Let the collection of all possible such sequences or paths be denoted Ω. A sample of size *n* is a partial sequence of the first *n* randomly selected letters in an ω, $\left\{x_{1},\cdots ,x_{n}\right\}$.

Let $p_{↓}=\left\{p_{\left(k\right)};k\geq 1\right\}$ and ${\hat{p}}_{↓}=\left\{{\hat{p}}_{\left(k\right)};k\geq 1\right\}$ be defined as above. It is to be specifically noted that the rearrangement of the observed relative frequencies, ${\hat{p}}_{↓}$, is performed independently based on the observed values of ${\hat{p}}_{k}$ for all k≥1, with no regard to the arrangement of the probabilities, $p_{↓}=\left\{p_{\left(k\right)};k\geq 1\right\}$. Consequently, the letter of which the relative frequency ${\hat{p}}_{\left(k\right)}$ is observed is not necessarily the same letter with which the probability $p_{\left(k\right)}$ is associated. This is, in fact, the essence of entropic perspective.

**Theorem** **2.** *For any p∈P, let $p_{↓}$, $\hat{p}$ and ${\hat{p}}_{↓}$ be defined as above. Then*

(19) $$\max_{k\geq 1}\left|{\hat{p}}_{\left(k\right)}-p_{\left(k\right)}\right|\overset{a.s.}{⟶}0.$$

A proof of Theorem 2 requires Lemmas 2 and 3 below.

**Lemma** **2.** *For any p∈P, let $\hat{p}$ be as defined above. Then*

(20) $$\max_{k\geq 1}\left|{\hat{p}}_{k}-p_{k}\right|\overset{a.s.}{⟶}0.$$

**Proof.**  For each *k*, by the strong law of large numbers, ${\hat{p}}_{k}\overset{a.s.}{⟶}p_{k}$ or equivalently $|{\hat{p}}_{k}-p_{k}|\overset{a.s}{⟶}0$. Let the collection of paths $\omega =\{x_{1},x_{2},\cdots \}$ in Ω that satisfies $\lim_{n\to \infty }\left|{\hat{p}}_{k}-p_{k}\right|=0$ be denoted as $\Omega_{k}\subseteq \Omega$. It follows that $P(\Omega_{k})=1$, that the complement of $\Omega_{k}$, $\Omega_{k}^{\prime }$, is of probability zero, that $\cup_{k\geq 1}\Omega_{k}^{\prime }$ is of probability zero, and that, letting $\Omega^{*}=\cap_{k\geq 1}\Omega_{k}$, $P\left(\Omega^{*}\right)=1-P\left(\cup_{k\geq 1}\Omega_{k}^{\prime }\right)=1$.

For each and every path $\omega \in \Omega^{*}$ and every *k*, $\lim_{n\to \infty }\left|{\hat{p}}_{k}-p_{k}\right|=0$. Note the fact that $|{\hat{p}}_{k}-p_{k}|\leq {\hat{p}}_{k}+p_{k}$ and, therefore, $\sum_{k\geq 1}\left|{\hat{p}}_{k}-p_{k}\right|\leq \sum_{k\geq 1}\left({\hat{p}}_{k}+p_{k}\right)=2$, by the bounded convergence theorem,

(21) $$\lim_{n\to \infty }\sum_{k\geq 1}|{\hat{p}}_{k}-p_{k}|=\sum_{k\geq 1}\lim_{n\to \infty }\left|{\hat{p}}_{k}-p_{k}\right|=0,$$

that is, $\sum_{k\geq 1}\left|{\hat{p}}_{k}-p_{k}\right|\overset{a.s.}{⟶}0$. By (21), the lemma follows from the fact that

$$\max_{k\geq 1}|{\hat{p}}_{k}-p_{k}|\leq \sum_{k\geq 1}\left|{\hat{p}}_{k}-p_{k}\right|\overset{a.s.}{⟶}0.$$

 □

Lemma 2 may be viewed as a version of the Glivenko–Cantelli theorem on countable alphabets with respect to observed data from a classical multinomial experiment. The uniformity of the convergence in (20) is of essential importance in the proof of Theorem 2, which is given below by way of Lemma 3.

**Lemma** **3.** *For each k≥1,*

(22) $${\hat{p}}_{\left(k\right)}-p_{\left(k\right)}\overset{a.s.}{⟶}0.$$

A proof of Lemma 3 is given in Appendix A. Let it be noted that Ω is the sample space of a perpetual multinomial *iid* sampling scheme on X under a probability distribution p∈P. Each path in Ω may be represented by $\left\{{\hat{p}}_{n};n\geq 1\right\}$ where ${\hat{p}}_{n}=\left\{{\hat{p}}_{k,n};k\geq 1\right\}$. For each such path $\{{\hat{p}}_{n};n\geq 1\}\in \Omega$, there exists a corresponding path $\left\{{\hat{p}}_{↓,n};n\geq 1\right\}$, which is the rearranged $\left\{{\hat{p}}_{n};n\geq 1\right\}$ over all *k* for every *n*. Let the total collection of all rearranged paths of Ω be denoted as $\Omega_{↓}$, and let the collection of all rearranged paths of $\Omega^{*}$ be denoted as $\Omega_{↓}^{*}$. It follows that $P\left(\Omega_{↓}^{*}\right)=P\left(\Omega^{*}\right)=1$. Lemma 3 states that, in each path of $\Omega_{↓}^{*}$, the *k* th component of ${\hat{p}}_{↓,n}$ converges to the *k* th component of $p_{↓}$, namely, $p_{\left(k\right)}$, for each *k*.

**Proof** **of** **Theorem** **2.** For any $\omega \in \Omega_{↓}^{*}$, note that $\sum_{k\geq 1}\left|{\hat{p}}_{\left(k\right)}-p_{\left(k\right)}\right|\leq 2$, by the bounded convergence theorem and Lemma 3, $\lim_{n\to \infty }\max_{k\geq 1}|{\hat{p}}_{\left(k\right)}-p_{\left(k\right)}|\leq \lim_{n\to \infty }\sum_{k\geq 1}|{\hat{p}}_{\left(k\right)}-p_{\left(k\right)}|=\sum_{k\geq 1}\lim_{n\to \infty }\left|{\hat{p}}_{\left(k\right)}-p_{\left(k\right)}\right|=0.$ The theorem follows the fact that $P(\Omega_{↓}^{*})=1$. □

Theorem 2 may be viewed as a version of the Glivenko–Cantelli theorem on countable alphabets with respect to observed data from an entropic multinomial experiment. Theorem 2 immediately implies almost sure convergence for estimators of several key quantities in classification procedures.

**Example** **4.** *${\hat{p}}_{\left(1\right)}\overset{a.s.}{\to }p_{\left(1\right)}$.*

**Example** **5.** Suppose that $p_{\left(1\right)}>p_{\left(2\right)}$, that is, there exists a unique letter in X, denoted $\ell_{0}$, associated with probability $p_{\left(1\right)}$. Then the probability of a correct classification, that is, $\ell_{0}={arg max}_{X}\left\{{\hat{p}}_{k};k\geq 1\right\}$, converges almost surely to one. This is so because, for any path in $\Omega_{↓}^{*}$ and any $\varepsilon <(p_{\left(1\right)}-p_{\left(2\right)})/2$, there exists an N such that, for any n>N, $|{\hat{p}}_{\left(1\right)}-p_{\left(1\right)}|<\varepsilon$ and $|{\hat{p}}_{\left(1\right)}-p_{\left(k\right)}|>\varepsilon$ for all k≥2.

The results of Examples 4 and 5 lend fundamental support for classification algorithms based on maximum observed frequency, used widely in exercises of modern data science, for example, decision trees, as mentioned in Example 3.

Many entropies of interest across a wide spectrum of studies are of the additive form, $H\left(p_{↓}\right)=\sum_{k\geq 1}g\left(p_{\left(k\right)}\right)h\left(p_{\left(k\right)}\right)$, where g(p)≥0 and h(p)≥0 are functions of p∈[0,1]. The almost-sure convergence of Theorem 2 may be passed on to the plug-in estimators of some such entropies by way of a rather trivial statement in the proposition below.

**Proposition** **1.** *Let $H\left(p_{↓}\right)=\sum_{k\geq 1}g\left(p_{\left(k\right)}\right)h\left(p_{\left(k\right)}\right)$ where g(p)≥0 and 0≤h(p)≤M for some M>0 are continuous functions of p∈I=[0,1]. Suppose that p∈P such that*

*1.*  *$\sum_{k\geq 1}g\left(p_{\left(k\right)}\right)<\infty$, and*
*2.*  *$\sum_{k\geq 1}g\left({\hat{p}}_{(k),n}\right)\overset{a.s.}{\to }\sum_{k\geq 1}g\left(p_{\left(k\right)}\right)$.*

*Then $H\left({\hat{p}}_{↓}\right)\overset{a.s.}{\to }H\left(p_{↓}\right)$.*

**Proof.**  Noting that $H\left(p_{↓}\right)=\sum_{k\geq 1}g\left(p_{\left(k\right)}\right)h\left(p_{\left(k\right)}\right)\leq M\sum_{k\geq 1}g\left(p_{\left(k\right)}\right)<\infty$, it follows by Conditions 1 and 2 that

(23) $$|H\left({\hat{p}}_{↓}\right)-H\left(p_{↓}\right)|\leq M\sum_{k\geq 1}g\left({\hat{p}}_{\left(k\right)}\right)+M\sum_{k\geq 1}g\left(p_{\left(k\right)}\right)<\infty .$$

Let $\Omega_{↓}^{**}\subseteq \Omega_{↓}$ be the total collection of paths such that Condition 2 holds. For each path, $\left\{{\hat{p}}_{n,↓};n\geq 1\right\}\in \Omega_{↓}^{**}$, by (23), the proposition follows by the bounded convergence theorem and the fact that $P(\Omega_{↓}^{**})=1$. □

**Example** **6.** Let $H\left(p_{↓}\right)=\sum_{k\geq 1}p_{\left(k\right)}^{s}{\left(1-p_{\left(k\right)}\right)}^{t}$ where s≥1 and t≥0 are two real constants. In the setup of Proposition 1, $h\left(p\right)={\left(1-p\right)}^{t}\leq 1$ on I=[0,1], and $g\left(p\right)=p^{s}$ satisfying $\sum_{k\geq 1}p_{\left(k\right)}^{s}\leq 1$ for all $p_{↓}\in P_{↓}$ without qualification, and therefore also for ${\hat{p}}_{↓}\in P_{↓}$, that is, $\sum_{k\geq 1}{\hat{p}}_{\left(k\right)}^{s}\leq 1$, which implies, by the bounded convergence theorem, $\sum_{k\geq 1}{\hat{p}}_{\left(k\right)}^{s}\to \sum_{k\geq 1}p_{\left(k\right)}^{s}$ along each and every path in $\Omega^{*}$. By Proposition 1, $\sum_{k\geq 1}{\hat{p}}_{k}^{s}{\left(1-{\hat{p}}_{\left(k\right)}\right)}^{t}\overset{a.s.}{\to }\sum_{k\geq 1}p_{k}^{s}{\left(1-p_{\left(k\right)}\right)}^{t}$. More specifically, when s and t take integers u≥1 and v≥0, the plug-in estimator of the generalized Simpson’s diversity index $H\left(p_{↓}\right)=\sum_{k\geq 1}p_{\left(k\right)}^{u}{\left(1-p_{\left(k\right)}\right)}^{v}$ (see [5,16]) converges almost surely.

Example 6 implies that the plug-in estimator of $H\left(p_{↓}\right)=\sum_{k\geq 1}p_{\left(k\right)}^{s}$ where s≥1 converges almost surely, which in turn implies that the plug-in estimators of members of the Rényi entropy family and the Tsallis entropy family converge almost surely for all $p_{↓}\in P_{↓}$ without qualification when α≥1. However, it is not known whether the plug-in estimators of the members of the families with α∈(0,1) converge almost surely when $p_{↓}\in P_{↓}$ without other qualification (also, see [17]).

**Example** **7.** The plug-in estimator of the Shannon entropy, $H\left(p_{↓}\right)=-\sum_{k\geq 1}p_{\left(k\right)}\ln p_{\left(k\right)}$, converges almost surely when $p_{↓}$ is such that $K=\sum_{k\geq 1}1_{\left[p_{\left(k\right)}>0\right]}<\infty$. In this case, even though −lnp is not bounded above on I=[0,1], $h\left(p\right)=-p^{\alpha }\ln p\leq 1/\left(\alpha e\right)$ is for any α∈(0,1). Writing $H\left(p_{↓}\right)=\sum_{k\geq 1}g\left(p_{\left(k\right)}\right)h\left(p_{\left(k\right)}\right)$ where $g\left(p\right)=p^{1-\alpha }$ and $h\left(p\right)=-p^{\alpha }\ln p$, it suffices to show $\sum_{k\geq 1}{\hat{p}}_{\left(k\right)}^{1-\alpha }$ converges almost surely. However, this is the case since, by Theorem 2, for every path $\left\{{\hat{p}}_{n,↓};n\geq 1\right\}\in \Omega_{↓}^{*}$, $\sum_{k\geq 1}{\hat{p}}_{\left(k\right)}^{1-\alpha }\to \sum_{k\geq 1}p_{\left(k\right)}^{1-\alpha }$, due to the fact that K<∞ and $P(\Omega_{↓}^{*})=1$.

It is not known whether the plug-in estimator of the Shannon entropy converges almost surely when $p_{↓}\in P_{↓}$ without further qualification.

The Shannon entropy has utilities across a wide spectrum of scientific investigations (see [18]). However, it is not finitely defined for all distributions in P. A family of the generalized Shannon entropies, for any $p_{↓}\in P_{↓}$, is proposed as follows:

(24) $$H_{m}\left(p_{↓}\right)=-\sum_{k\geq 1}\left(\frac{p_{\left(k\right)}^{m}}{\sum_{j\geq 1}p_{\left(j\right)}^{m}}\right)\ln\left(\frac{p_{\left(k\right)}^{m}}{\sum_{j\geq 1}p_{\left(j\right)}^{m}}\right)$$

in [19], where m≥1 is an integer. The Shannon entropy is a special family member corresponding to m=1. It may be verified that each member of the family, except the Shannon entropy, is finitely defined for all p∈P and offers all important utilities that the Shannon entropy offers, including the fact that the mutual information derived based on each member with m≥2 is zero if and only if the two underlying random elements are independent.

**Example** **8.** *The plug-in estimator of (24) converges almost surely for any $p_{↓}\in P_{↓}$ whenever m≥2. To see this, let it be first noted that the plug-in estimator of $-\sum_{k\geq 1}p_{k}^{m}\ln p_{k}$ converges almost surely. This fact follows from Proposition 1 with g(p)=p, $h\left(p\right)=-p^{m-1}\ln p$ which is uniformly bounded above on I=[0,1]. The claimed almost-sure convergence then follows the fact that, in the re-expression of (24) below,*

(25) $$H_{m}\left(p_{↓}\right)=\left(\frac{1}{\sum_{j\geq 1}p_{\left(j\right)}^{m}}\right)\left[\left(-m\sum_{k\geq 1}p_{\left(k\right)}^{m}\ln p_{\left(k\right)}\right)+\left(\sum_{k\geq 1}p_{\left(k\right)}^{m}\right)\left(\ln\sum_{j\geq 1}p_{\left(j\right)}^{m}\right)\right],$$

*and the fact that the plug-in estimator of each of the four series converges almost surely.*

## 5. Conclusions and Discussion

This article introduces a perspective termed entropic statistics. One of the motivations of the perspective is to accommodate probability experiments on sample spaces which may include outcomes that are known to exist (and therefore are prescribed) and those whose existence is not known (and therefore not prescribable). Such a framework allows statistical exploration into a general population with possibly infinitely many previously unobserved and unknown outcomes, or new discoveries. The key concept to foster such a framework is the label-independence, that is, all parameters and statistics do not depend of the labels of an alphabet as long as they are distinguishable. Consequently, in this article an array of label-independent objects are defined and termed entropic objects. In particular, a general entropy, entropic parameters, entropic statistics, entropic sample spaces, entropic probability distributions, and an entropic-moment-generating function are defined.

Based on the defined entropic objects, two basic theorems are established. Theorem 1 provides a characterization of the entropic probability distribution on the alphabet via the entropic-moment-generating function, and Theorem 2 establishes the almost-sure convergence of the entropic statistics to the entropic parameters and, hence, provides a foundational support to the entropic framework.

On the other hand, this article merely provides a few basic results in entropic statistics. On a broader spectrum, many other issues may be fruitfully considered on at least three fronts, namely, fundamental, probabilistic, and statistical. To begin with, the fundamental question of what constitutes entropy may be explored in many directions. One of the most cited sets of axioms is that discussed by Khinchin [20], under which the Shannon entropy is proved to be unique. However under slightly less restrictive axioms, many other entropies exist and enjoy almost all the desirable utilities of the Shannon entropy; for example, see [19]. The existing literature on generalization of entropy is extensive in physics and information theory; for example, see [21,22]. The collective effort to better understand what entropy is and how it may help to describe an underlying random system is ongoing. Further research in understanding generalized entropies and their implications could greatly enrich the framework of entropic statistics.

Entropy in general is often thought of as summary of a profile state, however measured numerically, of inner energy or chaos within a random system. As such, it is independent of any labeling systems, regardless of whether the state is observable or not. A key conceptual shift introduced in this article is from statistical inference on p (or a function of p) based on the multinomial frequencies Y to that on $p_{↓}$ (or a function of $p_{↓}$) based on the entropic frequencies $Y_{↓}$. Such a framework shift, by necessity or by choice, triggers a long array of basic probability and statistics questions, under different degrees of model restriction, ranging from parametric forms of $p_{k}=p\left(k,\theta \right)$ for some parameter θ to the nonparametric form, $\left\{p_{\left(k\right)};k\geq 1\right\}$. It may be interesting to note that even for the nonparametric form, there are several qualitatively different forms, that of a known $K=\sum_{k\geq 1}1_{\left[p_{\left(k\right)}>0\right]}<\infty$, that of an unknown $K=\sum_{k\geq 1}1_{\left[p_{\left(k\right)}>0\right]}<\infty$, and that of $K=\sum_{k\geq 1}1_{\left[p_{\left(k\right)}>0\right]}=\infty$. Each of these model classes could imply a very different stochastic behavior of $Y_{↓}$ as the sample size *n* increases. Even long before the notion of information entropy was coined by Shannon in [1], the behavior of $Y_{↓}$ had been discussed in the literature by, for example, Auerbach [23] and Zipf [24]. More recently, several articles [25,26] discussed domains of attraction in the total collection of all distributions on a countable alphabet by a tail index, $\tau_{n}=n\sum_{k\geq 1}p_{\left(k\right)}{\left(1-p_{\left(k\right)}\right)}^{n}$. Each domain characterizes the decay rate of the tail of the underlying entropic distribution and, in turn, dictates the rates of convergence of various statistical estimators of various entropies. Further advances on that front would enhance the understanding of probabilistic behavior of the entropic statistics and, in turn, the estimated entropies of interest.

In terms of statistical estimation, a large proportion of the existing literature mainly focuses on the Shannon entropy and variations of the plug-in estimators under various conditions, most of which are described and referenced in [14]. There are also non-plug-in estimators of different types, for example, the Bayes estimators [27,28,29], the hierarchical Bayes estimators [30], the James–Stein estimators [31], the coverage-adjusted estimators [32,33,34], and an unbiased estimator based on sequential data proposed by Montgomery-Smith and Schürmann. In general, the asymptotic distributions of the plug-in estimators and their variants seem to have been studied and described to some extent; for example, see [12,35,36,37,38]. However, it is fair to say that many, if not most, of the proposed estimators of various types have not yet been assigned asymptotic distributions. Any advances in that direction could much benefit applications of these estimators.

In short, the landscape of entropic statistics is quite porous in comparison to that of richly supported classical statistics. Many basic and important questions are yet to be answered, from the axiomatic foundation, to the definitions of basic elements, to the theoretical supporting architecture, and to the relevance in applications. However, the same said porosity also offers opportunities for interesting contemplation.

## Figures and Tables

**Table 1:** Confidence Levels of Simple Binary Classifier.

$p_{↓}$	n=3	n=5	n=7
(1/2,1/2)	0.5000	0.5000	0.5000
(2/3,1/3)	0.7407	0.7901	0.8267
(3/4,1/4)	0.8438	0.8965	0.9294
(4/5,1/5)	0.8960	0.9421	0.9667
(5/6,1/6)	0.9259	0.9645	0.9824

## Data Availability

This research involved no data.

## Appendix A

**Proof** **of** **Lemma** **3.** For clarity, the proof of (22) is given, respectively, in five progressively more general cases: (1) $p_{1}=1$; (2) $K=\sum_{k\geq 1}1_{\left[p_{k}>0\right]}<\infty$ and all positive $p_{k}$’s are distinct; (3) K<∞; (4) *K* is infinite and all positive $p_{k}$’s are distinct; (5) *K* is infinite. For notation simplicity in all cases, let it be assumed without loss of generality that $p=\{p_{k};k\geq 1\}$ is nonincreasingly arranged to begin with, that is, $p_{k}\geq p_{k+1}$ for every *k*. With this assumption, the only rearranged object is ${\hat{p}}_{↓}=\left\{{\hat{p}}_{\left(k\right)};k\geq 1\right\}$ with ${\hat{p}}_{\left(k\right)}\geq {\hat{p}}_{\left(k+1\right)}$ for every *k*. In Case 1, the statement of (22) is trivial. In Case 2, let $p_{0}=1$ and $p_{K+1}=0$. It follows that

$$1=p_{0}>p_{1}>p_{2}>\cdots >p_{k-1}>p_{k}>p_{k+1}>\cdots >p_{K}>p_{K+1}=0.$$

For each sequence $\omega \in \Omega^{*}$ as defined in the proof of Lemma 2, the uniformity of (20) implies that for any ε>0, there exists an *N* such that for all n>N, $-\varepsilon <{\hat{p}}_{k}-p_{k}<\varepsilon$ for all k≥1. Specifically, let

(A1) $$\varepsilon_{0}=\min\left\{\left(p_{k}-p_{k+1}\right)/2;1\leq k\leq K\right\}>0.$$

There exists an *N* such that for all n>N, $\max\{|{\hat{p}}_{k}-p_{k}\left|;1\leq k\leq K\right\}<\varepsilon_{0}$, which has the following two implications.

1. $\cap_{k=1}^{K}\left(p_{k}-\varepsilon_{0},p_{k}+\varepsilon_{0}\right)=\emptyset$, that is, $p_{k}\pm \varepsilon_{0}$ are disjoint for all k=1,…,K.
2. For every *k*, k=1,…,K, ${\hat{p}}_{k}\in p_{k}\pm \varepsilon_{0}$ and it is the only observed relative frequency in $p_{k}\pm \varepsilon_{0}$.

Combining the above two implications, it follows that ${\hat{p}}_{k}={\hat{p}}_{\left(k\right)}$, that is, $|{\hat{p}}_{\left(k\right)}-p_{k}|\to 0$, for every *k*, k=1,…,K. Since $\Omega^{*}$ is of probability one, (22) is established. In Case 3, it is allowed that several consecutive probabilities in $p=\{p_{k};1\leq k\leq K\}$, where $K=\sum_{k\geq 1}1\left[p_{k}>0\right]<\infty$, are identical. It follows that

$$1=p_{0}\geq p_{1}\geq p_{2}\geq \cdots \geq p_{k-1}\geq p_{k}\geq p_{k+1}\geq \cdots \geq p_{K}>p_{K+1}=0.$$

Noting that $p=\{p_{k};k\geq 1\}$ is a finite sequence of runs of identical values, collecting the first value in each run and retaining its index value, a subset of $\left\{p_{k};k\geq 1\right\}$ is obtained, namely, $\left\{p_{k_{i}};i=1,\ldots ,I\right\}$, where *I* is the number of distinct values in p. Let $r_{i}$ be the multiplicity of $p_{k_{i}}$ in p, i=1,…,I. It follows that

$$1=p_{0}=p_{k_{0}}>p_{k_{1}}>p_{k_{2}}>\cdots >p_{k_{K^{\prime }}}>p_{K+1}=0.$$

For each sequence $\omega \in \Omega^{*}$ as defined in the proof of Lemma 2, the uniformity of (20) implies that for any ε>0, there exists an *N* such that for all n>N, $-\varepsilon <{\hat{p}}_{k}-p_{k}<\varepsilon$ for all *k*, k=1,…,K. Specifically, let

(A2) $$\varepsilon_{1}=\min\left\{\left(p_{k_{i}}-p_{k_{i+1}}\right)/2;i=0,\ldots ,I\right\}>0$$

where $p_{k_{0}}=p_{0}=1$. There exists an $N_{1}$ such that for all $n>N_{1}$, $\max\{|{\hat{p}}_{k}-p_{k}\left|;k\geq 1\right\}<\varepsilon_{1}$, which has the following implications.

1. $\cap_{i=1}^{I}\left(p_{k_{i}}-\varepsilon_{1},p_{k_{i}}+\varepsilon_{1}\right)=\emptyset$, that is, $p_{k_{i}}\pm \varepsilon_{1}$ are disjoint for all *i*, i=1,…,I.
2. For every given *k*, and therefore an implied *i*, there are exactly $r_{i}$ relative frequencies among $\left\{{\hat{p}}_{k};1\leq k\leq K\right\}$ found in $p_{k_{i}}\pm \varepsilon_{1}$.

It then follows that, for each given *k*,

$$\min\left\{{\hat{p}}_{\left(k_{i}+j\right)};j=0,\ldots ,r_{i}-1\right\}\leq {\hat{p}}_{\left(k\right)}\leq \max\left\{{\hat{p}}_{\left(k_{i}+j\right)};j=0,\ldots ,r_{i}-1\right\},$$

and hence ${\hat{p}}_{\left(k\right)}\to p_{\left(k\right)}=p_{k}.$ Finally, (22) follows the fact that $P(\Omega^{*})=1$. In Case 4, $p_{k}>0$ for all k≥1 and all probabilities are distinct. Letting $p_{0}=1$ and $p_{\infty }=0$,

$$1=p_{0}>p_{1}>p_{2}>\cdots >p_{k-1}>p_{k}>p_{k+1}>\cdots >p_{\infty }=0.$$

For every fixed $k^{\prime }$ such that $p_{k^{\prime }}\in \left(0,1\right)$, let m≥1 be an integer such that

(A3) $$1-\sum_{k=1}^{m}p_{k}<p_{k^{\prime }+1},\mathrm{and}$$

(A4) $$m\geq k^{\prime }+1.$$

Such an *m* exists for any given p with an infinite *K* and a fixed $k^{\prime }\geq 1$. For each sequence $\omega \in \Omega^{*}$, as defined in the proof of Lemma 2, the uniformity of (20) implies that for any ε>0, there exists an *N* such that for all n>N, $-\varepsilon <{\hat{p}}_{k}-p_{k}<\varepsilon$ for all *k*, k≥1. Specifically, let

$$\varepsilon_{2}=\min\left\{\left(p_{k}-p_{k+1}\right)/2;k=0,\ldots ,m\right\}>0.$$

There exists an $N_{2}$ such that for all $n>N_{2}$, $\max\{|{\hat{p}}_{k}-p_{k}\left|;k\geq 1\right\}<\varepsilon_{2}$, which implies the following.

1. The first *m* probabilities of p, $p_{1},\cdots ,p_{m}$, are covered, respectively, by *m* disjoint intervals, $p_{k}\pm \varepsilon_{2}$, k=1,…,m.
2. The relative frequencies corresponding to $\left\{p_{1},\cdots ,p_{m}\right\}$, namely, $\left\{{\hat{p}}_{1},\cdots ,{\hat{p}}_{m}\right\}$, are also covered, respectively, by the same disjoint intervals, $p_{k}\pm \varepsilon_{2}$, k=1,…,m.

On the other hand, noting the strict inequality in (A3) and the fact that $k^{\prime }$ is a fixed integer, there exists a sufficiently small $\varepsilon_{3}$ such that

(A5) $$1-\sum_{k=1}^{m}p_{k}+m\varepsilon_{3}<p_{k^{\prime }+1}$$

or equivalently

(A6) $$1-\sum_{k=1}^{m}\left(p_{k}-\varepsilon_{3}\right)<p_{k^{\prime }+1}.$$

Let $\varepsilon_{4}=\min\left\{\varepsilon_{2},\varepsilon_{3}\right\}$. By Lemma 2, there exists an $N_{4}$ such that for all $n>N_{4}$,

(A7) $$p_{k}-\varepsilon_{4}<{\hat{p}}_{k}<p_{k}+\varepsilon_{4},$$

for all *k*, k=1,⋯,m, and that the updated (A5) and (A6) hold, namely,

$$1-\sum_{k=1}^{m}p_{k}+m\varepsilon_{4}<p_{k^{\prime }+1}$$

or, equivalently,

(A8) $$1-\sum_{k=1}^{m}\left(p_{k}-\varepsilon_{4}\right)<p_{k^{\prime }+1}.$$

That is, in each of the disjoint intervals of (A7), there is at least one relative frequency. In particular, ${\hat{p}}_{k}$ is covered in $\left(p_{k}-\varepsilon_{4},p_{k}+\varepsilon_{4}\right)$ for each *k*, $k=1,\ldots ,k^{\prime }<m$, by (A4). Next it is necessary to show that there may not be more than one relative frequency in $\left(p_{k}-\varepsilon_{4},p_{k}+\varepsilon_{4}\right)$ for each *k*, $k=1,\ldots ,k^{\prime }$. Toward that end, consider the total mass of 100% distributed among ${\hat{p}}_{k}$, k≥1, given *n*. From interval $\left(p_{1}-\varepsilon_{4},p_{1}+\varepsilon_{4}\right)$ to interval $\left(p_{m}-\varepsilon_{4},p_{m}+\varepsilon_{4}\right)$, the total collective mass covered is at least $\sum_{k=1}^{m}{\hat{p}}_{k}$; however, by (A7) and (A8),

$$\begin{array}{cc} \sum_{k=1}^{m}{\hat{p}}_{k}= & \sum_{k=1}^{m}\left({\hat{p}}_{k}+\varepsilon_{4}\right)-m\varepsilon_{4}>\sum_{k=1}^{m}p_{k}-m\varepsilon_{4}=\sum_{k=1}^{m}\left(p_{k}-\varepsilon_{4}\right)>1-p_{k^{\prime }+1} \end{array}$$

and the remainder of the mass is

(A9) $$\begin{array}{cc} 1-\sum_{k=1}^{m}{\hat{p}}_{k}< & p_{k^{\prime }+1}<p_{k^{\prime }+1}+\varepsilon_{4}. \end{array}$$

Regardless of the mass, $1-\sum_{k=1}^{m}{\hat{p}}_{k}$, on the left side of (A9) is allocated to one or more than one letter, other than those in $\left\{\ell_{1},\cdots ,\ell_{m}\right\}$, the corresponding ${\hat{p}}_{k}$, k≥m+1, could not possibly be sufficiently large to exceed $p_{k^{\prime }+1}+\varepsilon_{4}$, nor, therefore, $p_{k^{\prime }}-\varepsilon_{4}$. That implies that, along the path of that selected $\omega \in \Omega^{*}$, for any $n>N_{4}$, ${\hat{p}}_{k}$ and ${\hat{p}}_{k}$ alone is covered in $\left(p_{k}-\varepsilon_{4},p_{k}+\varepsilon_{4}\right)$ for *k*, $k=1,\ldots ,k^{\prime }$. This immediately implies that ${\hat{p}}_{k}={\hat{p}}_{\left(k\right)}$ for all *k*, $k=1,\ldots ,k^{\prime }$, and in particular ${\hat{p}}_{k^{\prime }}={\hat{p}}_{\left(k^{\prime }\right)}$. ${\hat{p}}_{\left(k^{\prime }\right)}\to p_{k^{\prime }}$ since ${\hat{p}}_{k^{\prime }}\to p_{k^{\prime }}$. Finally (22) follows the fact that $P(\Omega^{*})=1$. In Case 5, $p_{k}>0$ for all k≥1 but the probabilities in $p=\{p_{k};k\geq 1\}$ are allowed to have multiplicities. Letting $p_{0}=1$ and $p_{\infty }=0$,

(A10) $$1=p_{0}>p_{1}\geq p_{2}\geq \cdots p_{k}\geq \cdots >p_{\infty }=0.$$

$p=\{p_{k};k\geq 1\}$ has a special pattern: its maximum value runs for $r_{1}$ times; then its second largest value runs for $r_{2}$ times, and so on and so forth. In general, its *i* th largest value runs for $r_{i}$ times followed by a run of its i−1 st largest value. Collect the first value in each run and record its index, $k_{i}$, i≥1, resulting in a strictly decreasing subsequence, $\left\{p_{k_{i}};i\geq 1\right\}$. Letting $k_{0}=0$ and $k_{\infty }=\infty$,

$$1=p_{0}=p_{k_{0}}>p_{k_{1}}>p_{k_{2}}>\cdots p_{k_{i}}>\cdots >p_{k_{\infty }}=p_{\infty }=0.$$

Consequently, $p=\{p_{k};k\geq 1\}$ may be viewed as a sequence containing $p_{k_{i}}$ for i≥1 with $r_{i}-1$$p_{k_{i}}$s between $p_{k_{i}}$ and $p_{k_{i+1}}$. Given a value of *k*, say $k^{\prime }$, there is an $i^{\prime }$ such that $p_{k^{\prime }}=p_{k_{i^{\prime }}}$ and $k^{\prime }$ must be one of the values from the list $\left\{k_{i^{\prime }},k_{i^{\prime }}+1,\cdots ,k_{i^{\prime }}+r_{i^{\prime }}-1\right\}$, noting $p_{k_{i^{\prime }}+r_{i^{\prime }}}=p_{k_{i^{\prime }}+1}<p_{k^{\prime }}$. Let *m* be such that

(A11) $$1-\sum_{i=1}^{m}r_{i}p_{k_{i}}<p_{k_{i^{\prime }}+1},\mathrm{and}$$

(A12) $$\sum_{i=1}^{m}r_{i}\geq k_{i^{\prime }}+1.$$

Such an *m* exists for any given p and a fixed $k^{\prime }\geq 1$, which fixes an $i^{\prime }$. For each sequence $\omega \in \Omega^{*}$ as defined in the proof of Lemma 2, the uniformity of (20) implies that for any ε>0, there exists an *N* such that for all n>N, $-\varepsilon <{\hat{p}}_{k}-p_{k}<\varepsilon$ for all *k*, k≥1. Specifically let

$$\varepsilon_{5}=\min\left\{\left(p_{k_{i}}-p_{k_{i+1}}\right)/2;i=0,\ldots ,m\right\}>0.$$

There exists an $N_{5}$ such that for all $n>N_{5}$, $\max\{|{\hat{p}}_{k}-p_{k}\left|;k\geq 1\right\}<\varepsilon_{5}$, which has the following two implications.

1. The first $\sum_{i=1}^{m}r_{i}$ probabilities of p, $p_{1},\cdots ,p_{\sum_{i=1}^{m}r_{i}}$, are covered, respectively, by $k_{i^{\prime }}$ disjoint intervals, $p_{k_{i}}\pm \varepsilon_{5}$, $i=1,\ldots ,i^{\prime }$.
2. The relative frequencies corresponding to $\left\{p_{1},\cdots ,p_{\sum_{i=1}^{m}r_{i}}\right\}$, namely, $\left\{{\hat{p}}_{1},\cdots ,{\hat{p}}_{\sum_{i=1}^{m}r_{i}}\right\}$, are also covered, respectively, by the same disjoint intervals, $p_{k_{i}}\pm \varepsilon_{5}$, $i=1,\ldots ,i^{\prime }$.

On the other hand, noting the strict inequality in (A11) and the fact that $k^{\prime }$ is a fixed integer, there exists a sufficiently small $\varepsilon_{6}$ such that

(A13) $$1-\sum_{i=1}^{m}r_{i}p_{k_{i}}+\varepsilon_{6}\sum_{i=1}^{m}r_{i}<p_{k_{i^{\prime }}+1}$$

or, equivalently,

(A14) $$1-\sum_{i=1}^{m}r_{i}\left(p_{k_{i}}-\varepsilon_{6}\right)<p_{k_{i^{\prime }}+1}.$$

Let $\varepsilon_{7}=\min\left\{\varepsilon_{5},\varepsilon_{6}\right\}$. By Lemma 2, there exists an $N_{7}$ such that for all $n>N_{7}$, all relative frequencies sharing the same $p_{k_{i}}$, namely, ${\hat{p}}_{k_{i}},{\hat{p}}_{k_{i}+1},\cdots ,{\hat{p}}_{k_{i}+r_{i}-1}$, are found in

(A15) $$\left(p_{k_{i}}-\varepsilon_{7},p_{k_{i}}+\varepsilon_{7}\right)$$

for all *i*, i=1,⋯,m, and the updated (A13) and (A14) are

$$1-\sum_{i=1}^{m}r_{i}p_{k_{i}}+\varepsilon_{7}\sum_{i=1}^{m}r_{i}<p_{k_{i^{\prime }}+1}$$

or, equivalently,

(A16) $$1-\sum_{i=1}^{m}r_{i}\left(p_{k_{i}}-\varepsilon_{7}\right)<p_{k_{i^{\prime }}+1}.$$

That is, in each of the disjoint intervals of (A15), there are at least $r_{i}$ relative frequencies. In particular, the $r_{i}$ relative frequencies, $\left\{{\hat{p}}_{k_{i}},{\hat{p}}_{k_{i}+1},\cdots ,{\hat{p}}_{k_{i}+r_{i}-1}\right\}$, are covered in $\left(p_{k_{i}}-\varepsilon_{7},p_{k_{i}}+\varepsilon_{7}\right)$ for each *i*, $i=1,\ldots ,i^{\prime }\leq m$, by (A12). Next it necessary is to show that there may not be more than $r_{i}$ relative frequencies in $\left(p_{k_{i}}-\varepsilon_{7},p_{k_{i}}+\varepsilon_{7}\right)$ for each *i*, $i=1,\ldots ,i^{\prime }$. Toward that end, consider the total mass of 100% distributed among ${\hat{p}}_{k}$, k≥1, given *n*. From interval $\left(p_{1}-\varepsilon_{7},p_{1}+\varepsilon_{7}\right)$ to interval $\left(p_{\sum_{i=1}^{m}r_{i}}-\varepsilon_{7},p_{\sum_{i=1}^{m}r_{i}}+\varepsilon_{7}\right)$, the total collective mass covered is at least $\sum_{i=1}^{m}r_{i}{\hat{p}}_{k_{i}}$; however, by (A15) and (A16),

$$\begin{array}{cc} \sum_{i=1}^{m}r_{i}{\hat{p}}_{k_{i}}> & \sum_{i=1}^{m}r_{i}\left(p_{k_{i}}-\varepsilon_{7}\right)>1-p_{k_{i^{\prime }}+1} \end{array}$$

and the remainder of the mass is

(A17) $$\begin{array}{cc} 1-\sum_{k=1}^{m}{\hat{p}}_{k}< & p_{k_{i^{\prime }}+1}<p_{k_{i^{\prime }}+1}+\varepsilon_{7}. \end{array}$$

Regardless of if the mass on the left side of (A17) is allocated to one or more than one letter, other than those in $\left\{\ell_{1},\cdots ,\ell_{\sum_{i=1}^{m}r_{i}}\right\}$, the corresponding ${\hat{p}}_{k}$, $k\geq \sum_{i=1}^{m}r_{i}+1$, could not possibly be sufficiently large to exceed $p_{k_{i^{\prime }}+1}+\varepsilon_{7}$, nor, therefore, $p_{k^{\prime }}-\varepsilon_{7}$. That implies that, along the path of that selected $\omega \in \Omega^{*}$, for any $n>N_{7}$, $\left\{{\hat{p}}_{k_{i}},{\hat{p}}_{k_{i}+1},\cdots ,{\hat{p}}_{k_{i}+r_{i}-1}\right\}$ and only $\left\{{\hat{p}}_{k_{i}},{\hat{p}}_{k_{i}+1},\cdots ,{\hat{p}}_{k_{i}+r_{i}-1}\right\}$ are covered in $\left(p_{k_{i}}-\varepsilon_{7},p_{k_{i}}+\varepsilon_{7}\right)$ for *i*, $i=1,\ldots ,i^{\prime }$. This immediately implies that

1. $\left\{{\hat{p}}_{k_{i^{\prime }}},{\hat{p}}_{k_{i^{\prime }}+1},\cdots ,{\hat{p}}_{k_{i^{\prime }}+r_{i}-1}\right\}=\left\{{\hat{p}}_{\left(k_{i^{\prime }}\right)},{\hat{p}}_{\left(k_{i^{\prime }}+1\right)},\cdots ,{\hat{p}}_{\left(k_{i^{\prime }}+r_{i}-1\right)}\right\}$ but is not necessarily equal component-wise;
2. $|{\hat{p}}_{\left(k_{i^{\prime }}+j\right)}-p_{k^{\prime }}|<\varepsilon_{7}$ for all $j=0,1,\ldots ,r_{i^{\prime }}-1$;
3. In particular, $|{\hat{p}}_{\left(k^{\prime }\right)}-p_{k^{\prime }}|<\varepsilon_{7}$.

Finally (22) follows the fact that $P(\Omega^{*})=1$. □
